# Characterizing the role of the alternative NF-κB pathway in diffuse large B-cell lymphoma

**DOI:** 10.1186/1753-6561-7-S2-O2

**Published:** 2013-04-04

**Authors:** Daisuke Ito, Jaime F Modiano

**Affiliations:** 1 Department of Veterinary Clinical Sciences, University of Minnesota College of Veterinary Medicine, St. Paul, MN, USA; 2 Masonic Cancer Center, University of Minnesota, Minneapolis, MN, USA

## Background

Diffuse large B-cell lymphoma (DLBCL) is the most common type of lymphoma in humans and dogs and has similar biology and clinical behavior in both species. Therefore, comparative approaches in understanding the disease may be beneficial to both species. The deregulation of nuclear factor kappa B (NF-κB) pathway, composed of classical and alternative pathways, is important in the pathogenesis of DLBCL. However, studies have largely focused on the classical pathway and the role of the alternative pathway is incompletely understood. In this study, we characterize the alternative NF-κB pathway in DLBCL to test its potential as a therapeutic target.

## Materials and methods

The activation of NF-κB pathways was analyzed by the expression, nuclear translocation, and binding to the NF-κB oligonucleotide probe, of classical and alternative NF-κB proteins in primary dog DLBCL cells using western blotting and ***electrophoretic mobility shift assay**.*

## Results

We demonstrated for the first time that the alternative NF-κB pathway, as well as the classical NF-κB pathway, is recurrently activated in primary dog DLBCL cells**.** The pattern of NF-κB protein expression was similar to that observed in human DLBCL cells.

## Conclusions

We propose the alternative NF-κB pathway as a novel target for lymphoma therapies. We are currently analyzing the effect of small interfering RNAs targeting the alternative NF-κB pathway for cell proliferation/viability and changes in genome-wide gene expression using a RNA-sequencing technology. The results will provide new insights on the roles of the alternative NF-κB pathway to develop novel treatment strategies for human and dog DLBCL using comparative oncology approaches.

## Financial support

MAF First Award Grant D12CA-302 (DI) and the University of Minnesota Animal Cancer Care and Research Program Fund.

